# *pulver*: an R package for parallel ultra-rapid *p*-value computation for linear regression interaction terms

**DOI:** 10.1186/s12859-017-1838-y

**Published:** 2017-09-29

**Authors:** Sophie Molnos, Clemens Baumbach, Simone Wahl, Martina Müller-Nurasyid, Konstantin Strauch, Rui Wang-Sattler, Melanie Waldenberger, Thomas Meitinger, Jerzy Adamski, Gabi Kastenmüller, Karsten Suhre, Annette Peters, Harald Grallert, Fabian J. Theis, Christian Gieger

**Affiliations:** 1 Research Unit of Molecular Epidemiology, Helmholtz Zentrum München, Neuherberg, Germany; 2 Institute of Epidemiology II, Helmholtz Zentrum München, Neuherberg, Germany; 3 German Center for Diabetes Research (DZD), Neuherberg, Germany; 4 Department of Medicine I, University Hospital Grosshadern, Ludwig-Maximilians-Universität, Munich, Germany; 5 Institute of Genetic Epidemiology, Helmholtz Zentrum München, Neuherberg, Germany; 6 Chair of Genetic Epidemiology, IBE, Faculty of Medicine, LMU Munich, Munich, Germany; 7 DZHK (German Centre for Cardiovascular Research), Partner Site Munich Heart Alliance, Munich, Germany; 8 Institute of Human Genetics, Helmholtz Zentrum München, Neuherberg, Germany; 9 Institute of Human Genetics, Technische Universität München, Munich, Germany; 10 Genome Analysis Center, Helmholtz Zentrum München, Neuherberg, Germany; 11 Institute of Experimental Genetics, Technical University of Munich, Freising-Weihenstephan, Germany; 12 Institute of Bioinformatics and Systems Biology, Helmholtz Zentrum München, Neuherberg, Germany; 13 Department of Twins Research and Genetic Epidemiology, Kings College, London, UK; 14 Department of Biophysics and Physiology, Weill Cornell Medical College in Qatar, Doha, Qatar; 15 Institute of Computational Biology, Helmholtz Zentrum München, Neuherberg, Germany; 16 Department of Mathematics, Technische Universitat München, Garching, Germany

**Keywords:** Algorithm, Linear regression interaction term, SNP–CpG interaction, Software

## Abstract

**Background:**

Genome-wide association studies allow us to understand the genetics of complex diseases. Human metabolism provides information about the disease-causing mechanisms, so it is usual to investigate the associations between genetic variants and metabolite levels. However, only considering genetic variants and their effects on one trait ignores the possible interplay between different “omics” layers. Existing tools only consider single-nucleotide polymorphism (SNP)–SNP interactions, and no practical tool is available for large-scale investigations of the interactions between pairs of arbitrary quantitative variables.

**Results:**

We developed an R package called *pulver* to compute p-values for the interaction term in a very large number of linear regression models. Comparisons based on simulated data showed that *pulver* is much faster than the existing tools. This is achieved by using the correlation coefficient to test the null-hypothesis, which avoids the costly computation of inversions. Additional tricks are a rearrangement of the order, when iterating through the different “omics” layers, and implementing this algorithm in the fast programming language C++. Furthermore, we applied our algorithm to data from the German KORA study to investigate a real-world problem involving the interplay among DNA methylation, genetic variants, and metabolite levels.

**Conclusions:**

The *pulver* package is a convenient and rapid tool for screening huge numbers of linear regression models for significant interaction terms in arbitrary pairs of quantitative variables. *pulver* is written in R and C++, and can be downloaded freely from CRAN at https://cran.r-project.org/web/packages/pulver/.

**Electronic supplementary material:**

The online version of this article (10.1186/s12859-017-1838-y) contains supplementary material, which is available to authorized users.

## Background

Hundreds of genetic variants associated with complex human diseases and traits have been identified by genome-wide association studies (GWAS) [1–4]. However, most GWAS only considered univariate models with one outcome and one independent variable, thereby ignoring possible interactions between different quantitative “omics” data [5], such as DNA methylation, genetic variations, mRNA levels, or protein levels. For example, studies observed associations between specific epigenetic-genetic interactions and a phenotype [6–8]. The lack of publications analyzing genome-wide interactions may result because of the high computational cost of running linear regressions for all possible pairs of “omics” data. Understanding the interplay among different “omics” layers can provide important insights into biological pathways that underlie health and disease [9].

Previous interaction analyses in genome-wide studies mainly considered interactions between single-nucleotide polymorphisms (SNPs), which led to the development of several rapid analysis tools. For example, *BiForce* [10] is a stand-alone Java program that integrates bitwise computing with multithreaded parallelization; *SPHINX* [11] is a framework for genome-wide association mapping that finds SNPs and SNP–SNP interactions using a piecewise linear model; and *epiGPU* [12] calculates contingency table-based approximate tests using consumer-level graphics cards.

Several rapid programs are also available for calculating linear regressions without interaction terms. For example, *OmicABEL* [13] efficiently exploits the structure of the data but does not allow the inclusion of an interaction term. The R package *MatrixEQTL* [14] computes linear regressions very quickly based on matrix operations. This package also allows for testing for interaction between a set of independent variables and one fixed covariate. However, interactions between arbitrary pairs of quantitative covariates would require iteration over covariates, which is quite inefficient.

Thus, our R package called *pulver* is the first tool to allow the user to compute *p*-values for interaction terms in huge numbers of linear regressions in a practical amount of time. The acronym *pulver* denotes parallel ultra-rapid *p*-value computation for linear regression interaction terms.

We benchmarked the performance of our implemented method using simulated data. Furthermore, we applied our algorithm to “omics” data from the Cooperative Health Research in the Region of Augsburg (KORA) F4 study (DNA methylation, genetic variants, and metabolite levels).

KORA comprises a series of independent population-based epidemiological surveys and follow-up studies of participants living in the region of Augsburg, Southern Germany [15].

Access to the KORA data can be requested via the KORA.Passt System (https://helmholtz-muenchen.managed-otrs.com/otrs/customer.pl).

### Implementation

*pulver* computes *p*-values for the interaction term in a series of multiple linear regression models defined by covariate matrices *X* and *Z* and an outcome matrix Y, containing continuous data, e.g. metabolite levels, mRNA or proteomics data. In most cases the residuals from the phenotype adjusted for other parameters are used. All matrices must have equal number of rows, i.e., observations. For efficiency reasons, *pulver* does not adjust for additional covariates, instead the residuals from the phenotype adjusted for other parameters should be used.

### Linear regression analysis

For every combination of columns *x*, *y*, and *z* from matrices *X* , *Y*, and *Z*, *pulver* fits the following multiple linear regression model:

$$ y={\beta}_0+{\beta}_1\ x+{\beta}_2\ z+{\beta}_3\  xz+\varepsilon, \varepsilon \sim i.i.d.N\left(0,{\sigma}^2\right), $$

where *y* is the outcome variable, *x* and *z* are covariates, and *xz* is the interaction (product) of covariates *x* and *z*. All variables are quantitative. We need to test the null hypothesis *β*_3_ = 0 against the alternative hypothesis *β*_3_ ≠ 0. In particular, we are not interested in estimating the coefficients *β*_1_ and *β*_2_, which allows us to take a computational shortcut. By centering and orthogonalizing the variables, we can reduce the multiple linear regression problem into a simple linear regression without intercept. Thus, we can compute the Student’s *t*-test statistic for the coefficient *β*_3_ as a function of the Pearson’s correlation coefficient between *y* and the orthogonalized *xz*: $t=r\sqrt{DF/\left(1-{r}^2\right)}$, where *DF* is the degree of freedom. See the Additional file 1 for a more detailed derivation.

By computing the *t*-statistic based on the correlation coefficient, which has a very simple expression in the simplified model, we avoid fitting the entire model including estimating the coefficients *β*_1_ and *β*_2_. This is much more efficient because we are actually only interested in the interaction term.

### Avoiding redundant computations

**Fig. 1:**
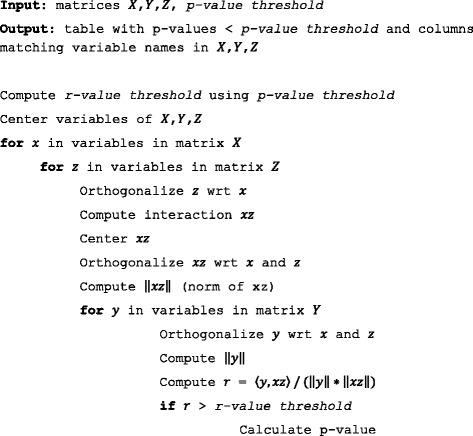
Pseudo-code of the *pulverize* function

Despite the computational shortcut, even more time can be saved by employing a sophisticated arrangement of the computations. The naïve approach would iterate through three nested for-loops, with one for each matrix, where all computations occur in the innermost loop. However, Fig. 1 shows that some computations can be moved out of the innermost loop to avoid redundant computations.

### Programming language and general information about the program

We implemented the algorithm in an R package [16] called *pulver*. Due to speed considerations, the core of the algorithm was implemented in C++. We used R version 3.3.1 and compiled the C++ code with gcc compiler version 4.4.7. To integrate C++ into R, we used the R package *Rcpp* [17] (version 0.12.7).

To determine whether C/Fortran could improve the performance compared to that of C++, we also implemented the algorithm using a combination of C and Fortran via R’s C interface.

We used OpenMP version 3.0 [18] to parallelize the middle loop. To minimize the amount of time required to coordinate parallel tasks, we inverted the order of matrices *X* and *Z* so that the middle loop could run over more variables than the outer loop, thereby maximizing the amount of work per thread.

To improve efficiency, the program does not allow covariates other than *x* and *z*. If additional covariates are required, the outcome *y* must be replaced by the residuals from the regression of *y* on the additional covariates. Missing values in the input matrices are replaced by the respective column mean.

Our *pulver* package can be used as a screening tool for scenarios where the number of models (number of variables in matrix *X* × number of variables in matrix *Y* × number of variables in matrix *Z*) is too large for conventional tools. By specifying a *p*-value threshold, the results can be limited to models with interaction term *p*-values below the threshold, thereby reducing the size of the output greatly. After the initial screening process, additional model characteristics for the significant models, e.g., effect estimates and standard errors, can be obtained with traditional methods such as R’s *lm* function.

The user can access *pulver’s* functionality via two functions: *pulverize* and *pulverize_all*. The *pulverize* function expects three numeric matrices and returns a table with p-values for models with interaction term *p*-values below the (optionally specified) *p*-value threshold. The wrapper function *pulverize_all* expects files with names containing *X*, *Y*, and *Z* matrices, calls *pulverize* to perform the actual computation, and returns a table in the same format as *pulverize*. The *pulverize_all* function is particularly useful if the matrices are too huge to be loaded all at the same time because of the computer memory restrictions. Thus, pulverize_all gets inputs as lists of file names containing the submatrices X, Y, and Z. *pulverize_all* iterates through these lists and subsequently loads matrices before calling the *pulverize*.

### Comparisons with other R tools for running linear regressions

**Fig. 2:**
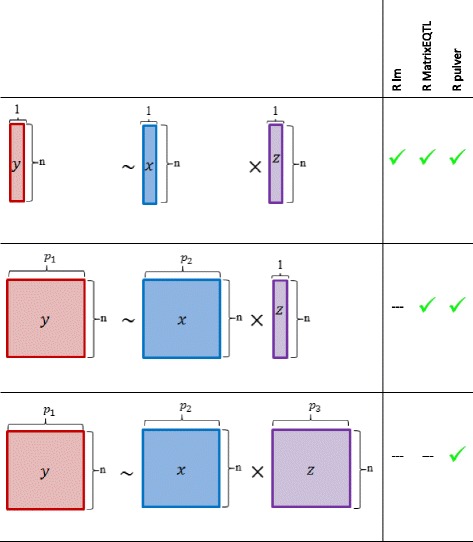
Comparison of different input types handled by the R tools *lm*, *MatrixEQTL*, and *pulver* for computation of the linear regression with interaction term. By the braces the dimensions of the matrices are depicted. The R’s build-in function *lm* can only compute the linear regression with interaction term using one variable with *n* observations per call. The R package *MatrixEQTL* is able to compute simultaneously the linear regression for each of *p*_1_ variables from the outcome matrix *Y* and the interaction term of the matrix *X* with *p*_2_ variables and the vector *Z*. In contrast, *pulver* in addition iterates through *p*_3_variables of the matrix *Z* and finally computes the linear regression for each column of matrices *Y* , *X* and *Z*

As illustrated in Fig. 2, the inputs for the interaction analysis can be vectors or matrices. Compared to other R tools such as *lm* and *MatrixEQTL pulver* is currently the only available option for users who want all the inputs to be matrices. It is possible to adapt other tools to all-matrix inputs, but the resulting code is not optimized for this use and will be too slow for practical purposes.

$$ {p}_1,{p}_2\ \mathrm{and}\ {p}_3\mathrm{are}\in \mathrm{\mathbb{N}}. $$

## Results

To benchmark the performance of *pulver* against other tools, we simulated *X*, *Y*, and *Z* matrices with different numbers of observations and variables.

We also applied *pulver* to real data from the KORA study.

### Performance comparison using simulated data

No other tool is specialized for the type of interaction analysis described above, so we compared the speed of our R package *pulver* with that of R’s built-in *lm* function and the R package *MatrixEQTL* [14] (version 2.1.1) (also see Fig. 2).

To ensure a fair comparison, we did not use the parallelization feature of pulverize because it is not available in R’s *lm* function or *MatrixEQTL*. However, parallelization is possible and it leads to significant speedups, although sublinear. For benchmarking purposes, each scenario was run 200 times using the R package *microbenchmark* (version 1.4–2.1, https://CRAN.R-project.org/package=microbenchmark) and the results were filtered with a *p*-value threshold of 0.05.

**Fig. 3:**
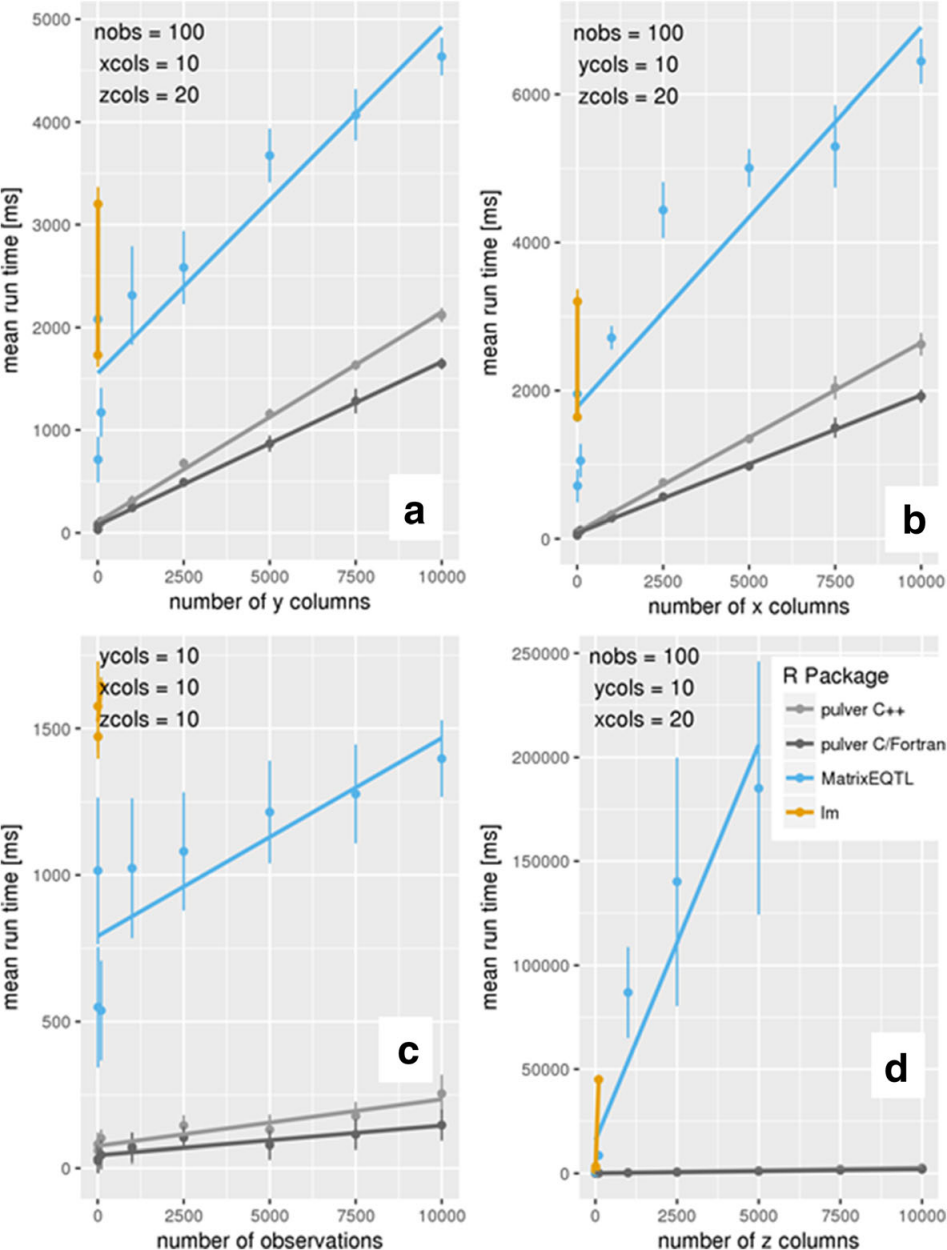
Mean run times and standard deviations for interaction analysis using R’s *lm* function, *MatrixEQTL*, and *pulver*. The execution times are in milliseconds. We fitted a line through the time points for each package. R’s *lm* function was very inefficient for this type of interaction analysis, and only the first two points are shown for every benchmark. Shown are four different panels (**a**-**d**). In panel **a** the number of columns of the matrix is set to 10, the matrix to 20 and the number of observations is set to 100, while the number of columns for the matrix is varied from 10 to 10,000. In panel **b** number of columns of the matrix is varied from 10 to 10,000 while the number of columns for the matrix is set to 10 column, the matrix to 20 column and number of observations is set to 100. In panel **c** the number of observations are varied from 10 to 10,000 while the number of columns for each matrix are fixed (all with 10 columns). In panel **d** number of columns of the matrix is varied from 10 to 10,000, while the number of columns of the matrix is set to 20, the matrix to 10 and the number of observations is set to 100

Figure 3 shows that *pulver* performed better than the alternatives in all the benchmarks. Note that the benchmark results obtained for the *lm* function were so slow that they could not be included in the chart.

In particular, for the benchmark where the number of variables in matrix *Z* was varied (see Fig. 3d), *pulver* outperformed the other methods by several orders of magnitudes, and the results obtained by *MatrixEQTL* could not be included in the chart. The poor performance of *MatrixEQTL* is because it can only handle one *Z* variable, which forced us to repeatedly call *MatrixEQTL* for every variable in the *Z* matrix. This type of iteration is known to be slow in R. The good performance of *pulver* with benchmark d is particularly notable because this benchmark reflects the intended user case for *pulver* where all input matrices contain many variables.

### Applying *pulver* to the analysis of real-world data

Metabolites are small molecules in blood whose concentrations can reflect the health status of humans [19]. Therefore, it is useful to investigate the potential effects of genetic and epigenetic factors on the concentrations of metabolites.

DNA methylation denotes the attachment of a methyl group to a DNA base. Methylation occurs mostly on the cytosine nucleotides preceding a guanine nucleotide, which are also called cytosine-phosphate-guanine (CpG) sites [20]. DNA methylation was measured using the Illumina InfiniumHumanMethylation450 BeadChip platform, which quantifies the relative methylation of CpG sites [21].

DNA methylation was measured in whole blood so it was based on a mixture of different cell types. We employed the method described by Houseman et al. [22] and adjusted for different proportions of cell types. Thus, CpG sites were represented by their residuals after regressing on age, sex, body mass index (BMI), Houseman variables, and the first 20 principal components of the principal component analysis control probes from 450 K Illumina arrays. The control probes were used to adjust for technical confounding, where they comprised the principal components from positive control probes, which were used as quality control for different data preparation and measurement steps.

Furthermore, to avoid false positives, all CpG sites listed by Chen et al. [23] as cross-reactive probes were removed. Cross-reactive probes bind to repetitive sequences or co-hybridize with alternate sequences that are highly homologous to the intended targets, which could lead to false signals.

In the KORA F4 study, genotyping was performed using the Affymetrix Axiom chip [24]. Genotyped SNPs were imputed with IMPUTE v2.3.0 using the 1000 Genomes reference panel.

Metabolite concentrations were measured using two different platforms: Biocrates (151 metabolites) and Metabolon (406 metabolites). Biocrates uses a kit-based, targeted quantitative by electrospray (liquid chromatography) – tandem mass spectrometry (ESI-(LC) MS/MS) method. A detailed description of the data was provided previously by Illig et al. [25]. Metabolon uses non-targeted, semi-quantitative liquid chromatography coupled with tandem mass spectrometry (LC-MS/MS) and GC-MS methods. The data were previously described in Suhre et al. [26].

Metabolites were represented by their Box–Cox transformed residuals after regressing on age, sex, and BMI. We used the R package *car* [27] to compute the Box–Cox transforms.

Initially, there were 345,372 CpG sites, 9,143,401 SNPs (coded as values between 0 and 2 according to an additive genetic model), and 557 metabolites in the dataset. Analyzing the complete data would have taken a very long time even with *pulver*.

Thus, to estimate the time required to analyze the whole dataset, we ran scenarios using all CpG sites, all metabolites, and different numbers of SNPs (100, 1000, 2000, 4000, and 5000), and extrapolated the runtime that would be required to analyze all SNPs. Due to time limitations, we ran each of the scenarios defined above only once. The estimated runtime required to analyze the complete dataset by parallelizing the work across 40 processors was 1.5 years.

Thus, we decided to only select SNPs that had previously known significant associations with at least one metabolite [1, 25]. We determined whether these signals became even stronger after adding an interaction effect between DNA methylation and SNPs.

To avoid an excessive number of false positives, the SNPs were also required to have a minor allele frequency greater than 0.05. We applied these filters separately to the Biocrates and Metabolon data. After filtering, we had 345,372 CpG sites, 117 SNPs, and 16 metabolites for Biocrates, with 345,372 CpG sites, 6406 SNPs, and 376 metabolites for Metabolon.

We were only interested in associations that remained significant after adjusting for multiple testing, so we used a *p*-value threshold of $\frac{0.05}{345372^{\ast }{117}^{\ast }16+{345372}^{\ast }{6406}^{\ast }376}={6.01}^{\ast }\ {10}^{-14}$ according to Bonferroni correction.

**Fig. 4:**
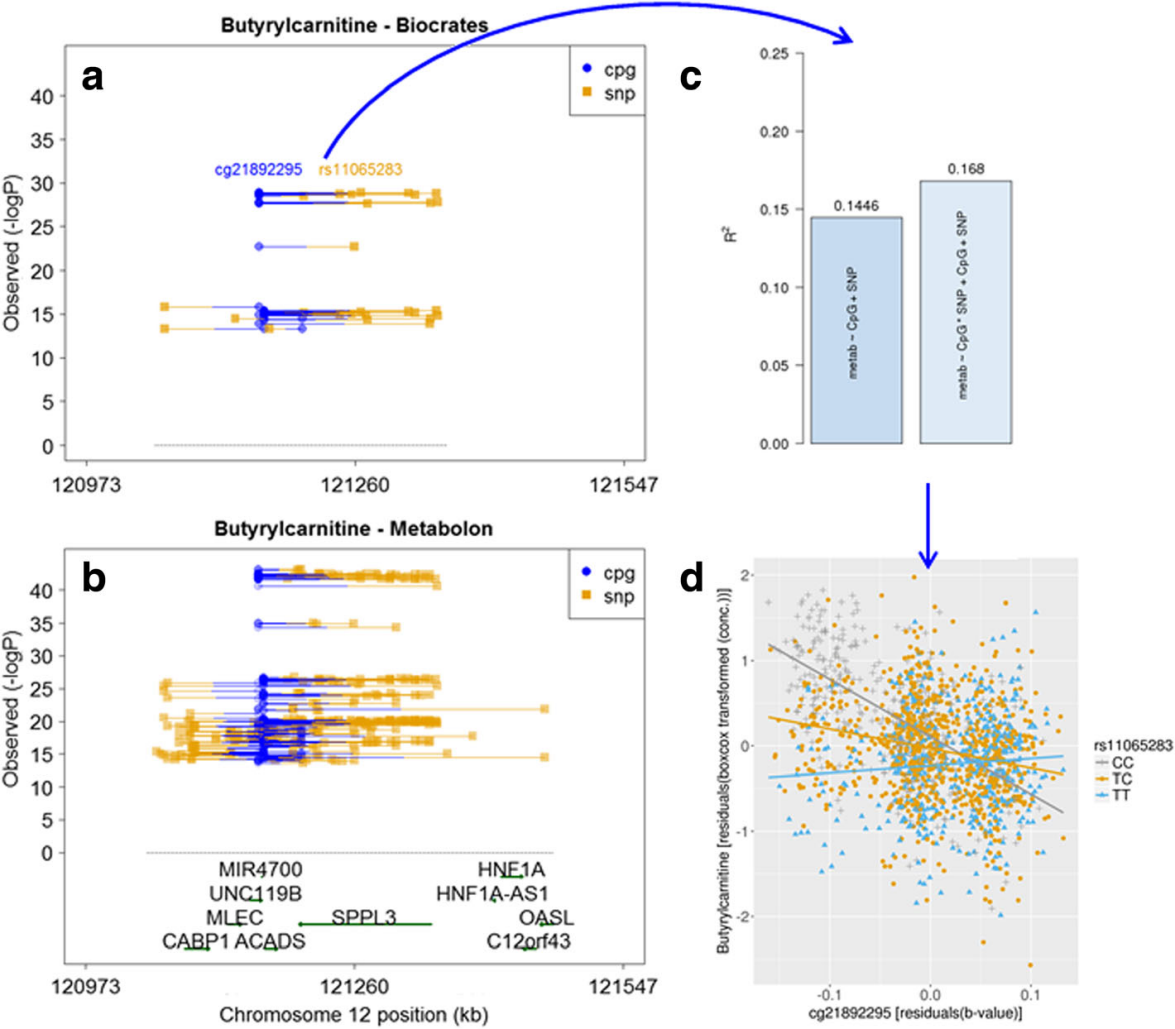
Regional plot with significant associations among SNPs (circles), CpGs (squares), and butyrylcarnitine for the Biocrates platform (**a**) and Metabolon platform (**b**). Interactions between SNPs and CpGs are visualized by lines connecting SNPs and CpGs. **c** Comparison of the adjusted coefficient of determination in the models with and without the interaction term. **d** Scatterplot of CpG site cg21892295 and metabolite butyrylcarnitine. Genotypes are color-coded

We found 27 significant associations for metabolites from the Biocrates platform (*p*-values ranging from 1.28^∗^ 10^−29^ to 5.17^∗^ 10^−14^) and 286 significant associations for metabolites from the Metabolon platform (*p*-values ranging from 1.15^∗^ 10^−42^ to 3.73^∗^ 10^−14^). All of the significant associations involved the metabolite butyrylcarnitine as well as SNPs and CpG sites on chromosome 12 in close proximity to the ACADS gene (see Fig. 4a and b). Figure 4c shows one of the significant results (SNP rs10840791, CpG site cg21892295, and metabolite butyrylcarnitine) to illustrate how the inclusion of an interaction term in the model increased the adjusted coefficient of determination,R^2^ (calculated using the summary.lm function in R).

The ACADS gene encodes the enzyme Acyl-CoA dehydrogenase, which uses butyrylcarnitine as a substrate [25], and previous studies have shown that SNPs and CpGs in this gene region are independently associated with butyrylcarnitine [1, 4, 25].

## Discussion

In the case where interaction terms need to be calculated for arbitrary pairs of variables, *pulver* performs far better than its competitors. The time savings are achieved by avoiding redundant calculations. Thus, computationally expensive *p*-values are only computed at the very end and only for results below a significance threshold determined using the (computationally cheap) Pearson’s correlation coefficient. To maximize the speedup, we recommend always specifying a *p*-value threshold and using *pulver* as a filter to find models with significant or near-significant interaction terms. If a *p*-value threshold is not specified, the time savings will be suboptimal and the number of results will be very high.

Thus, we recommend using a *p*-value threshold to adjust for multiple testing, such as Bonferroni correction, i.e. $\frac{0.05}{\mathrm{number}\  \mathrm{of}\  \mathrm{tests}}$ *.,* number of tests = number of columns in *X* × number of columns in *Y* × number of columns in *Z*.

The core algorithm of *pulver* was implemented in two languages namely, C++ and C/Fortran, to examine different performances due to programming languages. However, comparing the two different implementation of *pulver* reveals no striking differences. Thus, we continued to use the C++ version as it offered some useful implemented functions such as those implemented in the C++ Standard Library algorithms [28].

The package imputes missing values based on their column means. If this is not required, then we recommend using other more sophisticated methods, such as the *mice* package in R [29], in order to remove missing values before applying *pulver*.

*pulver* was developed as a screening tool to efficiently identify associations between the outcome, such as metabolite levels, and the interaction among two quantitative variables, such as CpG-SNP interaction. Once, significant associations are identified, other information regarding the fitted models, such as slope coefficients, standard errors, or residuals, can be computed in a second step using traditional tools.

## Conclusion

Our *pulver* package is currently the fastest implementation available for calculating *p*-values for the interaction term of two quantitative variables given a huge number of linear regression models. *Pulver* is part of a processing pipeline focused on interaction terms in linear regression models and its main value is allowing users to conduct comprehensive screenings that are beyond the capabilities of existing tools.

## Availability and requirements

Project name: pulver.

Project home page: https://cran.r-project.org/web/packages/pulver/index.html

Operating system(s): Platform independent.

Programming language: R, C++.

Other requirements: R 3.3.0 or higher.

License: GNU GPL.

Any restrictions to use by non-academics: None.

## Additional file

Additional file 1: Theory underlying pulver. This file describes the derivation of the t-value computed from the beta value divided by the standard error and the correlation value. (PDF 426 kb)

## Abbreviations

GWAS: Genome-wide association studies
SNP: Single-nucleotide polymorphism
