# Supplementary material for: pulver: an R package for parallel ultra-rapid p-value computation for linear regression interaction terms
Source: BMC Bioinformatics. 2017 Sep 29;18:429. doi: 10.1186/s12859-017-1838-y (PMC5622569; doi:10.1186/s12859-017-1838-y)
Supplement: Additional file 1: — Theory underlying pulver. This file describes the derivation of the t-value computed from the beta value divided by the standard error and the correlation value. (PDF 426 kb) [file 12859_2017_1838_MOESM1_ESM.pdf]

Theory underlying *pulver*

A general linear regression model is given as

$$y = X\beta + \epsilon, \quad \epsilon \sim N(0, \sigma^2 I).$$

Here $y^T = (y_1, y_2, \dots, y_n)$ is an $n \times 1$ vector, the dependent variable. X represents the $n \times (p+1)$ covariate matrix with corresponding $\beta^T = (\beta_0, \beta_1, \dots . \beta_p)$ a $(p+1) \times 1$ vector of unknown regression coefficients. The $n \times 1$ vector ϵ serves as error term has variance σ^2, and $\epsilon_1, \dots, \epsilon_n$ are independent and identical distributed (i.i.d.).

Let $X = \begin{pmatrix} 1 & x_1 & z_1 & w_1 = x_1 \cdot z_2 \\ 1 & x_2 & z_2 & w_2 = x_2 \cdot z_2 \\ \vdots & \vdots & \vdots & \vdots \\ 1 & x_n & z_n & w_n = x_n \cdot z_n \end{pmatrix}$, and the unknown regression coefficients $\beta^T = (\beta_0 \quad \beta_1 \quad \beta_2 \quad \beta_3)$. The then above general linear model reduces to the following linear regression model

$$y = \beta_0 + \beta_1 x + \beta_2 z + \beta_3 w + \epsilon, \quad \epsilon \sim N(0, \sigma^2),$$

with $\epsilon_1, \dots, \epsilon_n$ being independent and identical distributed ($i.i.d.$).

We want to test the null-hypothesis that $\beta_3 = 0$ against the alternative hypothesis that $\beta_3 \neq 0$, where β_3 is the regression coefficient of w. In order to eliminate the intercept β_0, we center all variables, such that $\sum_i y_i = \sum_i x_i = \sum_i z_i = \sum_i w_i = 0$, to obtain the following simplified regression model:

$$y = \beta_1 x + \beta_2 z + \beta_3 w + \epsilon, \qquad \epsilon \sim N(0, \sigma^2)\ i.i.d.$$

(For simplicity, we retain the notations from above for the simplified model (for variable names y, x, z, and w for the centered variables, regression coefficients, error term))

The vectors x, z and w span a subspace S of $\mathbb{R}^n$. The ordinary least-squares (OLS) estimates of $\hat{\beta}$ are found by minimizing the residual sum of squares over $y - X\beta$:

$$\hat{\beta} = \arg\min_\beta\ (y - X\beta)^T (y - X\beta).$$

Geometrically, this means that $\hat{\beta}_1$, $\hat{\beta}_2$, and $\hat{\beta}_3$ must be selected such that

$$y' = \hat{\beta}_1 x + \hat{\beta}_2 z + \hat{\beta}_3 w \qquad (1)$$

is the orthogonal projection of y onto S, the subspace spanned by w, x, and z. It can be shown that if x, y, and z form an orthogonal basis of S, the coefficients of the orthogonal projection y' of y onto S are given by

$$\hat{\beta}_1 = \frac{\langle y,x \rangle}{\langle x,x \rangle} \ , \ \hat{\beta}_2 = \frac{\langle y,z \rangle}{\langle z,z \rangle} \ , \ \hat{\beta}_3 = \frac{\langle y,w \rangle}{\langle w,w \rangle} \ [1].$$

Unlike the usual formula for computing OLS coefficient estimates ($\hat{\beta} = (X^T X)^{-1} X^T y$), this formula does not involve an expensive matrix inversion, but instead is easy and fast to compute.

In general, w, x, and z do not form an orthogonal basis, so we proceed as follows.

1. Create an orthogonal basis $v_1, v_2,$ and v_3 for S based on x, z, and w, respectively.
2. Compute y', the orthogonal projection of y onto S, using the orthogonal basis created in step 1.
3. Deduce the estimate of the regression coefficient for w from the regression coefficients for y'.
4. Compute the Student's *t*-test statistic to test $\beta_3 = 0$ as a function of the correlation coefficient r between y' and $\beta_3 v_3$.

1. Create an orthogonal basis for S

Let

$v_1 = x,$

$v_2 = z - proj(z, v_1),$ and

$v_3 = w - proj(w, v_1) - proj(w, v_2),$

where

$$proj(a, b) = \frac{\langle a, b \rangle}{\langle b, b \rangle} b$$

is the orthogonal projection of a onto b. The vectors v_1, v_2 and v_3 form an orthogonal basis of S. By construction, we clearly observe that v_1 is dependent on x only, v_2 is dependent on z and x and v_3 depends on $x, z,$ and w.

2. Orthogonally project y onto S

The orthogonal projection y' of y onto S has the form

$$y' = \beta_1' v_1 + \beta_2' v_2 + \beta_3' v_3 \qquad (2)$$

where

$$\beta_i' = \frac{\langle y, v_i \rangle}{\langle v_i, v_i \rangle} \quad (i = 1, 2, 3),$$

$$\text{with } \|a\| = \sqrt{\langle a, a\rangle},$$

$$\text{and } \langle a, b\rangle = \sum_{i=1}^{n} a_i b_i \text{ being the inner product of vectors } a \text{ and } b \text{ in } \mathbb{R}^n.$$

3. Deduce the estimate of w's regression coefficient

We want to estimate the regression coefficient β_3 of the vector w given in Equation 1 using Equation 2. The vector w occurs in v_3 but not in v_1 or v_2. This allows us to write y' as

$$\begin{aligned}
y' &= \beta_1' v_1 + \beta_2' v_2 + \beta_3' v_3 \\
&= \beta_1' v_1 + \beta_2' v_2 + \beta_3' \left(w - proj(w, v_1) - proj(w, v_2)\right) \\
&= \beta_1' v_1 + \beta_2' v_2 + \beta_3' w - \beta_3' proj(w, v_1) - \beta_3' proj(w, v_2) \\
&= \beta_3' w + \beta_1' v_1 + \beta_2' v_2 - \beta_3' proj(w, v_1) - \beta_3' proj(w, v_2) \\
&= \beta_3' w + \beta_1' v_1 + \beta_2' v_2 - \underbrace{\beta_3' \frac{\langle w, v_1\rangle}{\langle v_1, v_1\rangle}}_{scalar} v_1 - \underbrace{\beta_3' \frac{\langle w, v_2\rangle}{\langle v_2, v_2\rangle}}_{scalar} v_2 \\
&= \beta_3' w + c\left(\underbrace{v_1}_{c(x)}, \underbrace{v_2}_{c(x,z)}\right) \\
&= \beta_3' w + c(x, z)
\end{aligned}$$

where $c(\ldots)$ represents a linear combination of x or x and z, accordingly. This allows us to identify β_3, and we estimate the regression coefficient of w in Equation (1):

$$\beta_3 = \beta_3' = \frac{\langle y, v_3\rangle}{\langle v_3, v_3\rangle}.$$

4. Compute the Student's t-test statistic to test $\beta_3 = 0$ as a function of the correlation coefficient r between y' and $\beta_3 v_3$.

We want to show that the Student's t-test statistic usually used to test for $\beta_3 = 0$ in a linear regression model, with $t \geq t^*$ for significant threshold t^* can be computed using the Pearson's correlation coefficient r. The Pearson's correlation coefficient r between y' and v_3 (both centered) is computed as follows:

$$r = \frac{\sum_{i=1}^{N} y_i'^2 v_{3i}}{\|y'\| \|v_3\|} = \frac{\sum_{i=1}^{N} y_i'^2 v_{3i}}{\sqrt{\sum_{i=1}^{N} y_i'^2} \sqrt{\sum_{i=1}^{N} v_{3i}^2}}.$$

It then has to hold that $r \geq t^* \cdot \sqrt{\frac{1}{DF + t^{*2}}}$ if we want to reject the null-hypotesis.

The fact that $v_1, v_2,$ and v_3 are orthogonal means that β_3 is actually the OLS estimate of the regression coefficient r in the simple linear regression

$$y' = \beta_3 \ v_3 \ + \epsilon, \quad \epsilon \sim N(0, \sigma^2) \ i.i.d \qquad (3)$$

The Student's t –statistic to test for coefficient $\beta_3 = 0$ is given by

$$t = \frac{\beta_3}{se(\beta_3)}$$

and it has a Student's t distribution with $DF = n - 4$ degrees of freedom. Subtracting 4 results from the number of regression coefficients in the initial model and the estimated variance of ϵ: $\hat{\beta}_1, \hat{\beta}_2, \hat{\beta}_3, s^2$.

From the theory of simple linear regression, we know the following relationships (e.g., see Snedecor and Cochran 1967 [2], chapter 7.3, p. 175 ff.):

a) $\beta_3 = r \, \frac{se(y\prime)}{se(v_3)}$

b) $se(\beta_3) = s \,/ \sqrt{\sum_{i=1}^{n} v_{3}{}_i^2}$

c) $s^2 = \frac{1 - r^2}{DF} \sum_{i=1}^{N} y_i'^2$

d) $se(a) = \sqrt{\frac{1}{n-1} \sum_{i=1}^{n} a_i^2}$, with vector a in $\mathbb{R}^n$ and $\sum_i a_i = 0$,

where β_3 is the OLS estimate of Equation 3; $\mathrm{se}(y)$ and $\mathrm{se}(v_3)$ are the sample estimates of the standard deviations of y and v_3, respectively; $\mathrm{se}(\beta_3)$ is the estimate of the standard deviation of β_3; s^2 is the OLS estimate of σ^2, the variance of the error term ϵ; r is the Pearson's correlation coefficient of y and v_3; and DF is the degree of freedom.

After plugging Equations a–d into the formula for the Student's t, we obtain the following:

$$t = \frac{\beta_3}{se(\beta_3)}$$

$$= \frac{r\frac{se(y')}{se(v_3)}}{\frac{s}{\sqrt{\sum_{i=1}^{N} v_{3\,i}^2}}}$$

$$= \frac{r\, se(y')\sqrt{\sum_{i=1}^{N} v_{3\,i}^2}}{se(v_3\,)\sqrt{\frac{(1-r^2)}{DF}\sum_{i=1}^{N} y_i'^2}}$$

$$= \frac{r\sqrt{DF}}{\sqrt{(1-r^2)}} \cdot \frac{se(y')\sqrt{\sum_{i=1}^{n} v_{3\,i}^2}}{se(v_3)\sqrt{\sum_{i=1}^{n} y_i'^2}}$$

$$= \frac{r\sqrt{DF}}{\sqrt{(1-r^2)}} \cdot \frac{\sqrt{\frac{1}{n-1}\sum_{i=1}^{n} y_i'^2}\sqrt{\sum_{i=1}^{n} v_{3\,i}^2}}{\sqrt{\frac{1}{n-1}\sum_{i=1}^{n} v_{3\,i}^2}\sqrt{\sum_{i=1}^{n} y_i'^2}}$$

$$= \frac{r\sqrt{DF}}{\sqrt{(1-r^2)}}$$

1. Saville D, Wood GR, Statistical methods: The geometric approach. Springer Science & Business Media; 2012.
2. Snedecor, G. and W. Cochran, Statistical methods. 6th ed. Ames Iowa: University Press; 1967. p. 349-352.
